# Metacognition and Reading: Comparing Three Forms of Metacognition in Normally Developing Readers and Readers with Dyslexia

**DOI:** 10.1002/dys.1501

**Published:** 2015-08-03

**Authors:** Nicola Brunswick

**Affiliations:** Faculty of Psychology, University of BergenBergen, Norway

**Keywords:** metacognition, knowledge, strategies, experience, dyslexia

## Abstract

Metacognition refers to ‘cognition about cognition’ and includes metacognitive knowledge, strategies and experiences (Efklides, 2008; Flavell, 1979). Research on reading has shown that better readers demonstrate more metacognitive knowledge than poor readers (Baker & Beall, 2009), and that reading ability improves through strategy instruction (Gersten, Fuchs, Williams, & Baker, 2001). The current study is the first to specifically compare the three forms of metacognition in dyslexic (*N* = 22) versus normally developing readers (*N* = 22). Participants read two factual texts, with learning outcome measured by a memory task. Metacognitive knowledge and skills were assessed by self-report. Metacognitive experiences were measured by predictions of performance and judgments of learning. Individuals with dyslexia showed insight into their reading problems, but less general knowledge of how to approach text reading. They more often reported lack of available reading strategies, but groups did not differ in the use of deep and surface strategies. Learning outcome and mean ratings of predictions of performance and judgments of learning were lower in dyslexic readers, but not the accuracy with which metacognitive experiences predicted learning. Overall, the results indicate that dyslexic reading and spelling problems are not generally associated with lower levels of metacognitive knowledge, metacognitive strategies or sensitivity to metacognitive experiences in reading situations. @ 2015 The Authors. *Dyslexia* published by John Wiley & Sons Ltd.

There is a special focus in today's schools on students' ability to engage in self-regulation, defined as the extent to which learners are ‘metacognitively, motivationally and behaviorally active participants in their own learning process’ (Zimmerman, 1986, p. 308). One aspect of self-regulation is students' ability to regulate learning through metacognitive processes. Metacognition refers to ‘cognition about cognition’ and is involved in monitoring and control of various cognitive activities (Koriat, 2007; Metcalfe, 2000). A distinction is typically made between metacognitive knowledge, strategies and experiences (Efklides, 2008, 2011). Metacognitive knowledge (Flavell, 1979) refers to the individual's knowledge and understanding of their cognitive abilities, task requirements and appropriate strategies. Metacognitive strategies refer to the deliberate use of cognitive strategies to control cognition (Efklides, 2008). Metacognitive experiences are feelings, judgements and task-specific knowledge that reflect what the person is aware of and feels during task performance (Efklides, 2008). All three facets of metacognition are assumed to be important for students' learning.

Although learning requires self-regulatory abilities, it also often depends on students' reading skills. The simple view of reading states that reading comprehension is the product of decoding skills and language comprehension (Hoover & Gough, 1990). According to this model, proficient reading occurs when decoding is automatized, and all cognitive resources available can be used to understand the meaning of the text. However, if decoding is a bottleneck in text reading, more cognitive resources will be needed to read the words correctly, and less resource can be allocated to the various cognitive and metacognitive processes that are involved in text comprehension. From this perspective, it is interesting to study the relationship between reading ability and metacognitive mechanisms involved in self-regulation.

Differences in metacognitive knowledge about reading and strategy use have consistently been found between normally developing readers and readers with comprehension problems (e.g. Anderson & Ambruster, 1984; Baker & Beall, 2009; Roeschl-Heils, Schneider, & van Kraayenoord, 2003). The general finding is that poor comprehenders have less understanding of which reading strategies are appropriate in different reading situations. Anderson and Ambruster (1984), for example, reported that poor comprehenders tend to skim, reread, integrate information, plan ahead and make inferences to a lesser extent than more skilled readers. Findings also indicate that poor comprehenders experience difficulties in identifying the inconsistencies in a text (Baker & Beall, 2009; Snow, Burns, & Griffin, 1998). However, Corkett, Parrila, and Hein (2006) found that university students with a history of reading difficulties used strategies to the same or to a larger extent than other students. Research on metacognitive experiences in text comprehension has also showed contradictory findings (Maki & McGuire, 2002). Whereas some studies have found that poor readers are better at predicting future performance than good readers (Gillström & Rönnberg, 1994, 1995), others have found the opposite pattern (Garner, 1987; Maki & Berry, 1984), and yet others have found no differences (Maki & Swett, 1987; Pressley *et al*., 1987). The relationship between metacognitive facets and text comprehension has also been studied in normal populations and in intervention studies. Kolić-Vehovec, Zubković, and Pahljina-Reinić (2014) reported that individual differences in metacognitive knowledge of reading strategies were related to text comprehension within three different age groups. In a review of several intervention studies, Gersten *et al*. (2001) found that reading comprehension can be improved in students with learning disabilities through strategy instruction.

Although the importance of metacognition of normally developing readers and readers with poor comprehension skills have been studied extensively, less is known about the role of metacognition in text learning in readers with poor decoding skills, often defined as dyslexia. Dyslexia is characterized by difficulties with basic decoding, that is, accuracy and/or fluency, and poor spelling abilities (Lyon, Shaywitz, & Shaywitz, 2003; Vellutino, Fletcher, Snowling, & Scanlon, 2004).

Metacognitive monitoring and control are not the cause of the reading and spelling difficulties seen in readers with dyslexia. Nonetheless, it is important to obtain more knowledge about the relationship between dyslexia and metacognition. This is because of its proposed role in text comprehension, which can be a secondary consequence of dyslexic reading and spelling problems (Lyon *et al*., 2003). Kirby *et al*. (2008) suggested that the application of learning strategies and approaches to learning in dyslexic individuals can be understood as either a consequence of or a compensation for their fundamental reading problems. A similar framework may be proposed for the relationship between dyslexia and metacognition in general. On the one hand, when reading is not perfectly automatized but requires a certain degree of focal attention, resources available for metacognitive monitoring and control may potentially be reduced. Metacognitive difficulties could then be seen as a consequence of dyslexia (Roth, 2008). On the other hand, individuals with dyslexia may develop and make use of metacognitive abilities in text comprehension to compensate for their deficiency in basic reading. This follows from existing research demonstrating that dyslexic readers benefit from the use of cognitive and behavioural strategies, and that metacognitive strategies are particularly beneficial in text comprehension (Fidler & Everatt, 2012).

The purpose of our study was to compare three metacognitive facets in normal and dyslexic readers, in order to obtain more knowledge about metacognition in dyslexia. This required that we developed a procedure for measuring these three facets in the same study, which is rarely performed in metacognition research (Efklides, 2008). Metacognitive knowledge was assessed by a self-report questionnaire developed for this study, and metacognitive strategies were assessed by an existing questionnaire (Anmarkrud & Bråten, 2009). Metacognitive experiences were measured using established procedures from experimental metacognition research (Koriat, 2007), which have previously been applied to metacomprehension research (Maki & McGuire, 2002).

We address the following specific research questions:

1. Do dyslexic readers differ from normally developing readers in terms of self-reported metacognitive knowledge?
2. Do dyslexic readers differ from normally developing readers in terms of the self-reported use of metacognitive strategies in text reading?
3. Do dyslexic readers differ from normally developing readers in terms of metacognitive experiences related to text reading and their correspondence to learning outcome?

## Method

### Participants

Twenty-two students with dyslexia (11 women, 11 men, *M*_age_ = 18.68, age range: 18–22 years) and 22 normally developing readers (eight women, 14 men, *M*_age_ = 19.05, age range: 18–23 years) were recruited from 10 upper secondary schools in Hordaland county, Norway. Participants were recruited through an invitation forwarded by the school administration to normal readers and students with a documented diagnosis of dyslexia. To confirm that the groups differed with respect to reading and spelling ability but were comparable with respect to nonverbal ability, which is a typical criterion for distinguishing between normally developing readers and readers with dyslexia (Kirby *et al*., 2008), measures of reading, spelling and nonverbal ability were included. To assess whether the groups differed with respect to reading history, questions about childhood reading motivation and reading habits were also included (see Kirby *et al*., 2008 for a similar procedure). The participation was anonymous, and each participant was rewarded a gift card (NKR 150,-) after the test session.

### Materials

#### Nonverbal ability

Individual differences in nonverbal ability were measured with the standardized and normed *Naglieri Nonverbal Ability Test* (Naglieri, 2008), a nonverbal test that consists of a series of geometric matrices in which one part is missing, and where the task on each trial is to determine which of a set of five alternative elements is the correct one.

#### Reading and spelling ability

Word reading ability was measured by the reading speed subtest of The Reading and Spelling Test for College and University Students (Strømsø, Hagtvet, Lyster, & Rygvold, 1997). Participants were given 5 min to read a text silently, and as fast and accurately as possible. Reading speed was measured as words read per minute. This test also measured reading comprehension by asking the person, at regular intervals, to indicate which out of three words, written in brackets, would make the current sentence meaningful. Spelling ability was measured by the proofreading subtest from the same test battery. Here, the task was to read a text of 269 words, and detect as many (of max 30) inaccurate spellings patterns as possible within a time limit of 2 min.

#### Reading history

Childhood reading motivation was measured by 2 four-point Likert-type scale items asking for the interest in reading and being read for, with responses ranging from *Yes*, *to a large extent* to *No*, *not at all*. (Responses that fell within the additional categories *Did not read*/*Was not read for* and *Don*'*t know* were not included in the analysis in the succeeding text.) Childhood reading habits were measured by 2 five-point Likert-type scale items asking for the reported frequency of reading fiction, newspapers, magazines and webpages, with response alternatives ranging from *Daily* to *Never*.

#### Learning material

The learning material consisted of two Norwegian texts printed in a booklet of A4-size paper. Each text consisted of 1000 words/four pages written in a two-column layout, was divided into shorter sections with subheaders, and contained three pictures. The texts were modified versions of texts found on different websites. The topics of the texts were ‘asfalt’ and ‘hair transplantation’, chosen because we predicted the student sample to have limited prior knowledge of them. All participants read the texts in the same order and were given 5 min to read the two first pages and 5 min to read the two last pages (i.e. the total time limit of each text was then 10 min). Within the time frame of 5 min, they were given the opportunity to look back at the text for support within each reading session, meaning that they were in principle allowed to read the text as many times as possible.

To control for the possible influence of prior knowledge, topic interest and effort while reading, three additional questions were included. Before reading each of the two texts, participants were asked to indicate their prior knowledge of each topic, from *Very little* to *Very much*. After reading each of the texts, participants were asked to indicate how interesting they found the text, from *Very uninteresting* to *Very interesting*, and how much effort they had made when reading the text, from *Very little* to *Very much*. Responses were indicated on a six-point scale.

#### Metacognition

Metacognitive knowledge, metacognitive strategies and metacognitive experience were measured by self-report questionnaires.

The questionnaire measuring metacognitive knowledge was administered before text reading, and consisted of 15 six-point Likert-type scale items designed to reflect three categories of metacognitive knowledge, that is, knowledge of (a) oneself as a reader, (b) one's own reading skills compared with others and (c) reading as a strategy. All items are presented in the Appendix.

The questionnaire measuring metacognitive strategies consisted of 24 six-point Likert-type scale items formulated as statements (for more detailed information about this questionnaire, see Anmarkrud & Bråten, 2009). The items of interest were those 20 that reflected (a) deep learning strategies (nine items), (b) surface learning strategies (seven items) and (c) lack of learning strategies (four items). This questionnaire was administered to participants immediately after they had read the two texts. For each item, participants had to indicate the extent to which the statement described an activity they had engaged in while reading the two texts, with responses ranging from *Not at all* to *Very often*. Examples of items used in the strategy scale are ‘I tried to understand the content better by relating it to something I know’ (deep), ‘Now and then I stopped reading to think through or repeat what I had read’ (surface) and ‘I had difficulties understanding how to approach the text’ (lack).

Metacognitive experiences were measured as self-reported predictions of performance (PoP) measured in conjunction with text reading, and judgements of learning (JoL) measured in conjunction with recognition judgments.

For each text, PoPs were rated twice. Halfway through the text (i.e. after 5 min), and upon completion (i.e. after 10 min), they were asked to indicate how many questions they thought they would be able to answer if given eight questions concerning the piece of text they had just read.

Thirty-two factual questions (16 for each text) were presented twice, chronologically in the same order as the texts had been read. First, questions were presented without the opportunity to provide answers. Instead, participants were to indicate the likelihood that they would be able to recognize the correct answer to each question if presented among four alternatives, on a six-point scale from *Very unlikely* to *Very likely*. This rating is referred to as a JoL judgement (see Souchay & Isingrini, 2012).

Participants were then presented with the same questions in the same order, with four response alternatives for each question, and were asked to select the correct alternative for each question.

### Procedure

General instructions were given verbally and in writing at the start of the test session. After these instructions, participants were asked to sign a consent form prior to commencing testing. Participants were tested in groups (mixed sessions of dyslexic and normal readers) in a quiet room at their schools. All questions were always read out loud. Following the completion of the test session, participants were debriefed, and questions pertaining to the study were addressed. The total duration of the test session was approximately 2 h.

## Results

### Group Differences in Measures of Reading, Spelling and Nonverbal Ability

Of the 22 dyslexic readers, 16 reported having received the diagnosis in elementary school and six in secondary school. A series of *t*-tests comparing participants categorized as dyslexic versus normally developing readers (see Table 1) showed that there were no significant group differences with respect to nonverbal ability, *t*(36) = −0.84, *p* = 0.40, *r* = 0.14, and that the participants with dyslexia scored significantly lower than their normally developing peers on reading speed, *t*(42) = 2.21, *p* = 0.03, *r* = 0.32 spelling ability, *t*(42) = 3.98, *p* < 0.001, *r* = 0.52, and reading comprehension, *t*(42) = 2.60, *p* = 0.01, *r* = 0.37. Childhood reading motivation, measured as the reported interest in reading and being read for, was lower in dyslexic readers (*M* = 2.63, *SD* = 0.56) than in normally developing readers (*M* = 2.18, *SD* = 0.61), *t*(38) = 2.43, *p* = 0.02, *r* = 0.37. Childhood reading habits, measured in terms of the reported frequency of reading fiction, newspapers, magazines and webpages as a child, was significantly lower in dyslexic readers (*M* = 3.51, *SD* = 0.81) compared with normally developing readers (*M* = 2.97, *SD* = 0.96), *t*(42) = 2.04, *p* < 0.05, *r* = 0.30.

**Table 1:** Mean performances and standard deviations on all measures across dyslexic and normally developing readers

	Dyslexic readers		Normal readers		
			
	M	SD	M	SD	Sig.
NNAT	107.88	10.55	110.77	10.39	ns
Reading speed	543.77	190.96	692.18	250.55	*
Reading comprehension	10.95	3.62	14.55	5.37	**
Spelling	6.41	4.47	13.23	6.68	***
Learning outcome	15.45	4.32	19.59	4.70	**
MK intra	3.36	1.15	5.01	0.63	***
MK inter	3.42	1.11	4.28	0.60	**
MK strategy	4.55	0.52	5.08	0.58	**
Deep strategies	3.40	0.93	3.70	0.88	ns
Surface strategies	3.81	0.87	4.08	0.71	ns
Lack of strategies	2.98	0.95	2.41	0.75	*
PoP mean	15.64	5.56	18.68	3.15	*
Calibration bias	0.18	5.0	−0.91	5.93	ns
JoL mean	3.38	0.71	4.07	0.60	**
Gamma correlation	0.25	0.26	0.15	0.29	ns
Effort	8.1	2.02	8.50	1.41	ns
Interest	5.45	2.13	5.50	1.79	ns
Prior knowledge	3.91	1.51	3.59	1.74	ns

The Naglieri Nonverbal Ability Test (NNAT) is from Naglieri (2008).

SD, standard deviation; MK intra, metacognitive knowledge of oneself as a reader; MK inter, metacognitive knowledge of one's own reading skills compared with others; MK strategy, metacognitive knowledge of strategy use; PoP, predictions of performance; JoL, judgments of learning.

* *p* < 0.05.

** *p* < 0.01.

*** *p* < 0.001.

### Background Variables

There were no difference between groups in self-reported prior knowledge, *t*(42) = 0.65, *p* = 0.10, *r* = 0.10, effort, *t*(42) = 0.78, *p* = 0.44, *r* = 0.12, or interest, *t*(42) = 0.08, *p* = 0.94, *r* = 0.01.

### Learning Outcome

Learning outcome was measured as the total number of correct responses on the recognition task. Dyslexic readers showed lower performance than normally developing readers, *t*(42) = 3.04, *p* = 0.004, *r* = 0.42.

### Metacognitive Knowledge

Separate sum scores were calculated for the three categories of metacognitive knowledge. For each subscale, we assessed the internal consistency by calculating Cronbach's α, which reflects the average correlation between the different items within each scale. Internal consistency reliability was high for metacognitive knowledge of oneself as a reader (α = 0.81), and of one's own reading skills compared with others (α = 0.84), showing that participants responded consistently within these two subscales. However, it was relatively low for the subscale assessing reading strategies (α = 0.50), indicating that participants responded less consistently on these items. Dyslexic readers had lower scores than normally developing readers on items reflecting metacognitive knowledge of oneself as a reader, *t*(42) = 5.87, *p* < 0.001, *r* = 0.67, one's own reading skills compared with others, *t*(42) = 3.20, *p* = 0.003, *r* = 0.44, and reading strategies, *t*(42) = 3.16, *p* = 0.003, *r* = 0.44.

### Metacognitive Strategies

Separate sum scores were calculated for the three categories of metacognitive strategies. Internal consistency reliability (Cronbach's α) was high for surface strategies (α = 0.68), deep strategies (α = 0.81), and lack of strategies (α = 0.73), showing that participants responded consistently within all three subscales. There were no group differences in the use of surface strategies, *t*(42) = 1.14, *p* = 0.26, *r* = 0.17, or deep strategies, *t*(42) = 1.09, *p* = 0.28, *r* = 0.17. However, dyslexic readers showed higher scores on lack of strategy, *t*(42) = 2.20, *p* = 0.03, *r* = 0.32.

### Metacognitive Experiences

Dyslexic readers had lower mean PoP compared with normally developing readers, *t*(42) = 2.23, *p* = 0.03, *r* = 0.33. They also had lower mean JoL, *t*(42) = 3.48, *p* = 0.001, *r* = 0.47. For each participant, we calculated a calibration bias score, that is, the difference between the total PoP and total learning outcome, with a positive score indicating overconfidence and a negative score indicating underconfidence. Groups did not differ with respect to calibration bias, *t*(42) = 0.66, *p* = 0.10. For each participant, we also calculated a gamma correlation on the relationship between JoL and recognition accuracy. There were no differences between the groups in terms of their gamma scores, *t*(42) = 1.20, *p* = 0.24, *r* = 0.18.

## Discussion

The present study investigated metacognitive knowledge, metacognitive strategies and metacognitive experiences in readers with dyslexia and normally developing readers. The dyslexic subgroup consisted of individuals who had received their diagnosis in elementary or secondary school. As expected, our results regarding reading and spelling ability, and nonverbal ability indicated that the dyslexic sample currently experienced reading and spelling difficulties but were comparable with normal developing readers on nonverbal ability. In addition, results regarding childhood reading history confirmed that the students with dyslexia had a relatively long history of reading difficulties. This is in line with the results of Kirby *et al*. (2008), who found that dyslexic readers differed from normally developing readers in terms of their scores on the Adult Reading History Questionnaire – Revised (Lefly & Pennington, 2000).

Dyslexic readers differed from normally developing readers in terms of all three forms of self-reported metacognitive knowledge. More specifically, own reading abilities were rated as lower by dyslexic readers. When asked to compare their reading skills and reading motivation with that of their peers, dyslexic readers also reported lower levels of reading ability. These findings are in line with what is generally known about dyslexic adults' insight into their own reading difficulties (Riddick, Sterling, Farmer, & Morgan, 1999). Knowledge of reading strategies was also lower in the dyslexic subsample. These results are with previous studies on individuals with comprehension problems (Anderson & Ambruster compatible, 1984; Baker & Beall, 2009; Roeschl-Heils *et al*., 2003), and with the results of Kolić-Vehovec *et al*. (2014), who showed that text comprehension was positively correlated with individual differences in metacognitive knowledge of strategy use. However, it should be noted that the internal consistency reliability (Cronbach's α) was low for this subscale in the present study. A possible reason is that our questions measuring knowledge of strategies may have reflected different subcategories of strategies, for example, background knowledge, overview of different parts of the texts and identifying a deeper structure of the text. If people's knowledge of each of these strategies is partly independent, it is perhaps not surprising that the internal consistency reliability was not higher. Future studies should therefore try to identify and measure knowledge of different subcategories of such strategies.

Even though dyslexic readers reported less knowledge of strategies, groups did not differ in terms of their self-reported tendency to apply deep and surface reading strategies during text reading. This apparent inconsistency may reflect that the two sets of items differ in their degree of specificity, with the questions concerning knowledge of strategies being more general and therefore perhaps more likely to reflect people's general understanding of reading strategies, which may or may not correspond to actual strategy use during text reading. Of course, it might be the case that dyslexic readers have less general knowledge of reading strategies, even though they are equally likely to apply these strategies during reading. However, one could also argue that whether or not one agrees with the various statements assessing strategies in general may depend on one's understanding of the role of task and context variables in strategy choice, which could differ between dyslexic and normally developing readers. The items that we used to measure metacognitive knowledge of strategies were designed to be general and context-free on purpose – in order to capture people's global knowledge of reading strategies. However, future studies should consider including items that contain more context information (e.g. about the type of text and amount of time available), even though questions would still not relate to specific strategies applied during reading of a specific text.

Our finding that dyslexic readers reported using deep and surface strategies during text reading equally often as normally developing readers is consistent with the findings of Kirby *et al*. (2008), who found that strategy use among dyslexic readers was even more frequent than among normally developing readers, which can be seen as a form of compensation. It should be noted that strategy use may be influenced by teacher instruction and/or support, both among dyslexic and normally developing readers. We did not control for this variable in our study, because we considered that this could not be adequately measured by students' own self-report. However, future studies that apply longitudinal designs and/or that use teachers as informants should consider including teacher instruction/support as a variable. It should be noted that dyslexic readers reported a higher tendency to experience difficulties in knowing which strategy to apply than did normally developing readers. This may indicate that dyslexic readers more often felt uncertain about how to approach the text. Especially when text reading is time limited, as was the case in our study, this may become particularly evident. In the future, self-report measures of metacognitive strategies as those used in our study could be supplemented by other types of data, for example, eye-tracking, which would provide a more precise measure of the relationship between self-report and actual strategy use.

Dyslexic readers showed lower performance on our measure of learning outcome but did not differ from normal developing readers with respect to prior knowledge, topic interest or effort invested in reading. Dyslexic readers also reported lower mean PoP and JoL than normally developing readers, which is not surprising given what we know about dyslexic students' insight into their own reading difficulties (Riddick *et al*., 1999). It is more interesting that they did not differ with respect to the degree of correspondence between metacognitive experiences and learning outcome. This was also the case for calibration scores, which is a measure of absolute metacomprehension accuracy that according to Maki, Shields, Wheeler, and Zacchilli (2005) should be more sensitive to individual differences in reading ability than measures of relative metacomprehension, of which gamma correlation is an example. Both sets of findings indicate that dyslexic readers are equally sensitive to metacognitive experiences during text reading as normally developing readers, because a lower predicted performance/memory is in line with the observed lower level of actual learning outcome. These results indicate that dyslexic readers have metacognitive insight into their own difficulties and are able to adjust their expectations regarding text comprehension and learning in line with their actual reading skills.

In general, our findings do not indicate that impaired metacognition is a consequence of poor decoding ability due to limits of focal attention (Roth, 2008). Even though lower metacognitive knowledge of strategies and a higher tendency to report difficulties in strategy application could be seen as a consequence of dyslexia, the lack of a difference in online metacognitive experiences and deep/surface strategy use goes against this hypothesis. This is because metacognitive experiences and strategies during reading and retrieval are more likely than other forms of metacognition to compete for the same resources as reading because they occur in direct conjunction with it. Moreover, because dyslexic readers were not better than normally developing readers in terms of strategy use and the accuracy of metacognitive experiences, our results alone cannot be used to support the hypothesis that dyslexics compensate for their difficulties with reading and spelling by developing better metacognitive abilities. To demonstrate that the identical performance level reflected compensation in our dyslexic sample, our results would need to be supplemented by longitudinal data.

In a controlled laboratory-based study, one will always have to weigh considerations regarding experimental control versus ecological validity against each other. In the current study, such considerations concerned the design of the reading situation (e.g. text complexity, available time and possibility of re-reading text), as well as the way in which metacognitive feelings were measured (e.g. PoP measurement required interrupting reading). Even though we have aimed to balance these two concerns against each other, there is always a risk that the reading situation may have been perceived as artificial, and that our measures of metacognitive experiences did not capture the whole range of such experiences as they manifest in everyday reading situations.

## Appendix

Questionnaire items measuring metacognitive knowledge of oneself as a reader (1–5), of one's reading skills compared with others (6–10) and of reading strategies (11–15), translated from Norwegian. End points were labelled ‘I totally disagree/I surely agree’ (items 1, 2, 3, 4, 5, 11, 12, 13, 14 and 15); Much poorer/Much better (items 6, 7 and 9); Much less/Much more (item 8); Much lower/Much higher (item 10).

1. I have problems reading individual words in texts.
2. I find it difficult to read fast.
3. I often have problems understanding the content when I read a text.
4. When I encounter a new text, I expect to be able to learn its content.
5. I am often motivated for reading.
6. How would you consider your reading speed compared with your classmates?
7. How would you consider your reading comprehension compared with your classmates?
8. How much do you think that you read in your spare time compared with your classmates?
9. When your class is given a new text, how well do you expect to learn its content compared with your classmates?
10. How would you consider your motivation for reading compared with your classmates?
11. Having background knowledge about a topic can make it easier to understand a text that is concerned with the topic.
12. Comprehension of a text will improve if you actively try to get an overview of central parts of the text.
13. When you encounter a new text, the best thing to do is always to look for headings/pictures because this will help you understand the text better.
14. In order to improve comprehension, it can be a good idea to occasionally stop reading, and to think about what you have just read.
15. If you have problems seeing the connection between different parts/ideas of a text, this does not reduce text comprehension.
